# Resilience of Breeding Boreal Waterbirds to Harsh Wintering Conditions: Could Climate Warming Smooth Population Declines?

**DOI:** 10.1002/ece3.73718

**Published:** 2026-05-27

**Authors:** Hannu Pöysä, Esa Lammi, Veli‐Matti Väänänen

**Affiliations:** ^1^ Department of Environmental and Biological Sciences University of Eastern Finland Joensuu Finland; ^2^ Natural Resources Institute Finland, Natural Resources Joensuu Finland; ^3^ Environmental Planning ENVIRO Espoo Finland; ^4^ Department of Forest Sciences University of Helsinki Helsinki Finland

**Keywords:** breeding waterbirds, climate change, extreme events, long‐term population trends, population dynamics, population resilience

## Abstract

Due to global climate change, winters have become milder and the ice season in lakes and other aquatic systems shorter across the Northern Hemisphere. Consequently, wintering conditions of open water‐dependent waterbirds have become milder. While winter warming‐caused changes in the distribution of wintering waterbirds have been documented for several species, it is unclear how warming of winters is reflected in population dynamics and long‐term trends of waterbirds in northern breeding areas. We used population count data and studied resilience (resistance to cold winters and recovery thereafter) of 15 waterbird species breeding in European boreal lakes to harsh wintering conditions during 1977–2022. Our aim was to assess the possibility that climate warming could smooth observed waterbird population declines caused by other anthropogenic stressors. In general, wintering conditions had only marginal impact on the dynamics (growth rate) of waterbird populations, except in the Common Coot ( *Fulica atra* ), in which cold winters affected population growth rate negatively. Even though population growth rate of most species was relatively resistant to cold winters, population trajectories of six species showed evidence of increase after a period of three consecutive exceptionally cold winters, suggesting high recovery rate. We found no evidence of association between resistance to cold winters and body size and species' thermal niches in their wintering ranges or between recovery rate and life history variables (clutch size and age at 1st reproduction). Nor did we find evidence of association between resistance to cold winters and long‐term population trend. Our results suggest that most waterbird species do not benefit from warming winters because their breeding populations appeared to be relatively unresponsive to variation in winter weather conditions. Hence, warmer winters may not provide a mechanism that could mitigate negative impacts of other anthropogenic stressors on breeding populations of waterbirds in northern Europe.

## Graphical abstract

Due to global climate change, wintering conditions of open water‐dependent waterbirds have become milder in central‐western Europe. We found that breeding populations of most waterbird species in northern Europe are resistant to cold winters, and this pattern was not associated with long‐term population trends. Results suggest that breeding populations of waterbirds in northern Europe may not benefit from warmer winters.

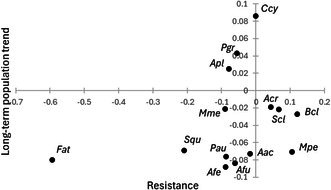

## 1 Introduction

Global climate change has been recognized as one of the main threats to biological systems at various organizational levels across the world (Parmesan and Yohe 2003; Root et al. 2003; Wiens and Zelinka 2024). Impacts of increasing mean temperatures and more frequently occurring heat extremes on the performance of individuals and distribution of species have been studied extensively (Parmesan 2006; Martínez‐De León and Thakur 2024). Both climatic phenomena have generally been recognized as threats to biological systems (Sabater et al. 2023; Martínez‐De León and Thakur 2024; Kotz et al. 2025), although some systems may benefit from increasing temperatures (Wu and Zhao 2024). While increasing mean temperatures and more frequently occurring heat extremes have been in focus, climate warming‐induced changes in the other end of the temperature gradient, viz. decrease of the magnitude and frequency of cold extremes, and their impacts on biological systems have received far less attention. Moreover, while such research has been done, the focus has been on assessing negative impacts (Williams et al. 2015; Reeve et al. 2023). To achieve a more comprehensive understanding of how biological systems respond to current climate change we need to study the whole range of impacts (negative and positive) of climate warming on the performance of species' populations.

One influential outcome of climate warming in the Northern Hemisphere is that the ice season in lakes and other aquatic systems has shortened, lakes in some regions having lost the ice season altogether (Benson et al. 2012; Sharma et al. 2019, 2021; Woolway et al. 2020). This has far‐reaching consequences for species and biological processes in aquatic ecosystems (Hampton et al. 2024), including waterbirds. For example, due to climate warming, the wintering conditions of waterbirds in inland lakes of western and central Europe have become milder, the occurrence of ice‐free winters being predicted to increase dramatically in the coming few decades (Sharma et al. 2019). The ice season has become shorter and the annual maximum ice extent smaller also in the Baltic Sea (HELCOM 2021), an important wintering area for several waterbird species (Skov et al. 2011; Nilsson and Hermansson 2021). Due to these climate warming‐caused changes in the ice conditions in the wintering areas, the winter distribution of many species in the Baltic Sea has shifted towards north and northeast (Lehikoinen et al. 2013; Pavón‐Jordán et al. 2019). Similarly, the occurrence and abundance of waterbirds wintering in inland wetlands in southern Sweden have increased with winter temperature (Gaget et al. 2025). As persistence of warm winter weather in northwestern Europe has increased in recent decades (Spanjers et al. 2025), it is increasingly important to know how warming winters affect breeding populations of waterbirds in northern Europe.

Waterbirds typically respond to exceptional cold spells in the wintering areas by moving to more benign areas with open water (Ridgill and Fox 1990; Keller et al. 2009; Gourlay‐Larour et al. 2012; Schummer et al. 2010; Meissner 2025), although the response may be weaker in late‐winter due to approaching spring migration (Masto et al. 2022). Even though movements are advantageous in terms of avoiding mortality due to cold weather, such weather‐induced movements may cause extra mortality. For example, moving farther south in Europe might increase hunting mortality because hunting pressure there is higher (Brides et al. 2017). In addition, preferred cold‐weather refuges may get overcrowded (Musilova et al. 2015), possibly increasing density dependent mortality. Indeed, analyses based on ringing recovery data suggest for many species that mortality increases during periods of abnormally cold weather (Ridgill and Fox 1990). Cavé and Visser (1985) in turn found that the survival of resident Common Coots ( *Fulica atra* ) decreased with increasing winter severity in the lake Westeinderplassen, The Netherlands. Direct evidence of winter severity‐caused mortality also exists for several waterbird species (Suter and van Eerden 1992; Blake‐Bradshaw et al. 2024). Finally, negative impacts of cold wintering conditions on breeding numbers of resident waterbird species have been reported from the UK (Tirozzi et al. 2024).

It is thus reasonable to expect that exceptionally cold weather conditions in the wintering areas affect negatively breeding numbers of waterbirds in northern European communities. However, analyses of effects of wintering conditions on breeding numbers of waterbirds in northern Europe are largely missing. Kauppinen and Väänänen (1999) studied effects of weather conditions in the wintering areas on breeding numbers of 12 waterbird species at lakes in central Finland during 1984–1995. They found support for winter severity effect only in the Common Goldeneye ( *Bucephala clangula* ): breeding numbers decreased with increasing winter severity index (maximum ice cover in the Baltic Sea). However, the time period in that analysis was relatively short, only 12 years. Pavón‐Jordán et al. (2017) analyzed a more extensive and longer dataset from Finland (a subset of national waterbird monitoring data from 1986 to 2015) and found that the combined abundance of 17 waterbird species breeding in Finland was positively associated with mild weather conditions (positive North Atlantic Oscillation index) in the wintering areas. Unfortunately, the study design was not at species‐specific level, preventing comparisons between species in their response to winter weather conditions. Without knowing the response of breeding populations of individual species to winter severity, it is difficult to predict how future climate warming will affect their population trajectories. Such species‐specific information is badly needed because species differ in conservation status; for example, from the 17 species included in Pavón‐Jordán et al. (2017), three species have been classified vulnerable and four species near threatened at the European level (BirdLife International 2021), two species out of these seven being classified also globally vulnerable (BirdLife International 2025).

Resilience to disturbances, such as those caused by extreme temperatures, is a fundamentally important property of populations and ecological communities to cope with global change (Capdevila et al. 2020; Martínez‐De León and Thakur 2024; Vitousek et al. 2025). At the population level, resilience is generally measured as change in population size and is characterized by two components: resistance (the degree of population decrease after a disturbance, strong decrease meaning low resistance) and recovery (the degree of population increase after the disturbance‐caused low) (Capdevila et al. 2020; Martínez‐De León and Thakur 2024). Species often differ in terms of both resistance and recovery, depending for example, on differences in life‐history strategies (Capdevila et al. 2020; Martínez‐De León and Thakur 2024). Therefore, to understand and predict responses of species and communities to impacts of climate change, it is important to study species‐specific resilience to varying climatic conditions and traits that may explain between‐species differences in resilience.

Here, we studied the resilience of populations of migratory waterbirds breeding in northern Europe (Finland) to exceptionally cold weather in their wintering areas in central‐western Europe. We addressed the following main study questions (SQ). First, how strong an overall driver are weather conditions in the wintering areas of the dynamics of breeding populations in northern Europe (SQ 1)? Second, how does the population growth rate of species respond to extreme cold winters (resistance; SQ 2) and how do populations recover from possible lows caused by extreme cold winters (SQ 3)? Considering that species may not show similar responses to wintering conditions (Masto et al. 2022), we studied if differences among species in resilience to harsh weather conditions in the wintering areas are associated with differences in species life history traits and winter thermal affinity (SQ 4)? In addition, we studied if species‐specific resistance to cold wintering conditions is associated with long‐term population trends (SQ 5). Specifically, because winters have become milder and assuming that species with weak resistance to cold wintering conditions would have benefitted relatively more from the warming winters, we predicted that species with weak resistance to harsh wintering conditions should have relatively more positive (or less negative) long‐term population trends than species with high resistance to harsh wintering conditions. With answering these questions, we aim to assess the possibility that, due to milder wintering conditions, climate warming could alleviate negative impacts of other anthropogenic stressors on populations of waterbirds breeding in boreal Europe. Indeed, while populations of some large waterfowl species such as geese and swans have been increasing in Europe (Fox et al. 2010; Holopainen et al. 2022), breeding numbers of waterbirds in boreal Europe are generally declining (Lehikoinen et al. 2016; Elmberg et al. 2020; Holopainen et al. 2024; Piha et al. 2025), underlining the need for a species‐level approach to deepen our understanding of climate change impacts on waterbird populations.

## 2 Materials and Methods

### 2.1 Winter Severity Data

**FIGURE 1:**
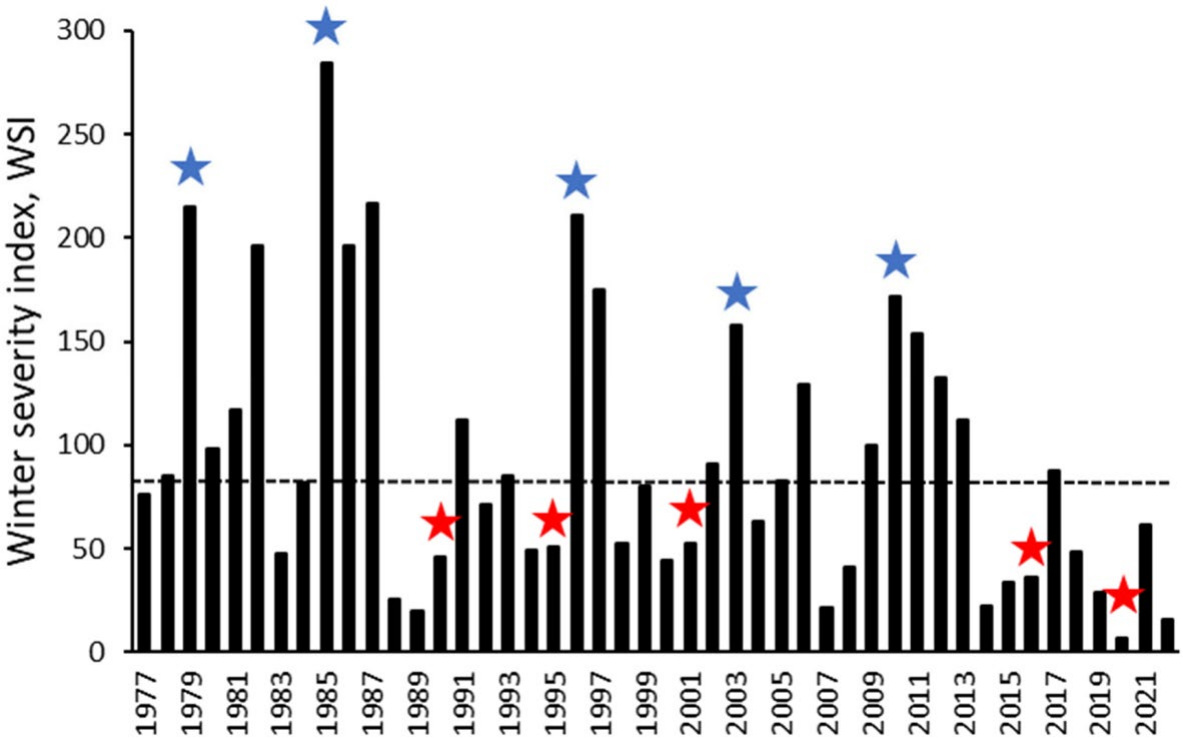
Winter severity index (WSI) in 1976/1977–2021/2022 for the main wintering area of waterbirds breeding in Finland. WSI is calculated as mean of the sums of mean daily temperatures that were below zero between 1 December and 28 February at six weather stations in central‐western Europe (higher WSI values equal with colder winters). Dashed horizontal line indicates mean for the period 1991–2020. Blue stars indicate five coldest winters and red stars five mildest winters (see ‘Materials and Methods’, ‘Winter Severity Data’ for criteria used to select the coldest and mildest winters).

We selected six weather stations in central‐western Europe (Malmö, southern Sweden; Groningen, The Netherlands; Berlin‐Dahlem, northeastern Germany; Strasbourg‐Entzheim, northeastern France; Munich, southern Germany; Zurich‐Fluntern, Switzerland; Table S1, Figure S1) to represent the main wintering areas of waterbirds breeding in Finland as revealed by winter‐time ring recoveries of birds ringed in Finland (Saurola et al. 2013). For these stations, we downloaded the mean daily temperatures for December, January, and February 1976–2022 from www.ecad.eu (Klein Tank et al. 2002) and calculated the Hellman index (Hellmann 1918) as the sum of mean daily temperatures that were below zero between 1 December and 28 February (winter severity index ‘WSI’, calculated as mean of the sums from the six stations, Figure 1; higher WSI values equal with colder winters; see also Pöysä et al. 2019; Pöysä 2024). WSI in central‐western Europe is correlated with corresponding WSI in southern Europe (*r* = 0.861, *p* < 0.001, *n* = 46), although winters in southern Europe are much milder (see Table S1 and Figure S2). WSI has been found to be useful when studying for example, effects of harsh wintering conditions on movements of waterbirds in the European wintering areas (Ridgill and Fox 1990). The WSI (central‐western Europe) data was used in the analyses in the following ways to answer the first three study questions outlined in the Introduction (SQ 1–3; see ‘Bird Population Data’ and ‘Statistical Analyses’ below for further data and methodological details). First, we used WSI as a continuous independent variable to study the overall effect of winter severity on population dynamics (growth rate) of each species using long‐term population time series data combined from different communities for the whole study period 1977–2022 (SQ 1). Second, because time series of population abundances typically are noisy, making the detection of impacts of extrinsic (e.g., winter severity) and intrinsic (e.g., density dependence) drivers on population dynamics difficult (e.g., Brook and Bradshaw 2006; Gebreyohannes and Houlahan 2024), we compared also impacts of extreme winters (cold vs. mild) on population growth rate, providing a more effective test to detect possible negative impacts of harsh winters on population growth rates. To that end, we selected five coldest and five mildest winters, with the criterion that each of the coldest and mildest winters was preceded by at least two mild winters; this criterion was to standardize the wintering conditions in the preceding years for the cold and mild winters that were in focus. With this criterion, the five coldest winters were 1978/1979, 1984/1985, 1995/1996, 2002/2003 and 2009/2010, and the five mildest winters were 1989/1990, 1994/1995, 2000/2001, 2015/2016 and 2019/2020 (Figure 1). With this setting we studied the response of population growth rate of each species to extreme cold winters (SQ 2). Third, there were three consecutive exceptionally cold winters in the mid‐1980s, viz. 1984/1985, 1985/1986 and 1986/1987, followed by several years of relatively mild winters (Figure 1). This provided a unique opportunity to study recovery of breeding numbers after a period of harsh winters (SQ 3).

### 2.2 Bird Population Data

We used time series of breeding waterbirds in local communities of 36 eutrophic lakes scattered across southern Finland (Figure S1), the longest continuous lake‐ and species‐specific time series covering the whole study period 1977–2022 (Table S2). We have used the time series earlier for other purposes, and details of bird census methods and data quality checking can be found in the earlier articles (Pöysä and Linkola 2021; Pöysä et al. 2023). The time series were not of the same length (i.e., each of the time series did not cover the whole study period 1977–2022) and some time series had missing years (bird censuses were not done). In addition, in some lake‐ and species‐specific time series, there were many zero years (bird census was done but a given species was not observed). For these reasons, data from a given time series were included only if suitable to answer a given study question as follows. To answer the first study question (SQ 1), we used all continuous (i.e., no missing years) time series that did have ≤ 20% of years with pair number = 0 (i.e., no observations of breeding pairs for a given species in bird censuses for that year) and no more than two consecutive years with pair number = 0. To answer the second study question (SQ 2), we used time series that provided data to calculate population growth rate (see ‘Statistical Analyses’ for calculation of population growth rate) for at least one of the years following the five coldest winters (see ‘Winter Severity Data’); if pair number was zero in both of the successive years (e.g., in 1978 and 1979) the data were not considered suitable to calculate population growth rate. These population growth rates were compared with those calculated for years following the five mildest winters (see ‘Winter Severity Data’), with the same criteria for data inclusion as for the coldest winters. We added 1 to the annual pair numbers of each time series to avoid problems with ‘zero’ years when calculating population growth rates to answer SQ 1 and SQ 2. To answer the third study question (SQ 3), we used time series that provided data to study possible recovery of pair numbers after the three consecutive extreme cold winters (see above). To that end, only time series that provided data for each year in 1987–1991 were included (i.e., no missing years but could include years with pair number = 0). The time series (annual pair numbers) were standardized (mean = 0, variance = 1) for each lake and species. We assumed that, if a given species responded to cold wintering conditions in the first place, its numbers were driven to a low by the three consecutive exceptionally cold winters, after which a population recovery started. This assumption was reasonable; for example, Kauppinen and Väänänen (1999) showed that, after the severe winter of 1984/1985 the total population of waterfowl breeding in their study area decreased by 16% in 1985 and by 26% from 1984 to 1986, remaining at the 1986 level (lowest during the study period 1984–1995) also after the severe winter 1986/87 (see figure 1 in Kauppinen and Väänänen 1999). A period of five years (1987–1991) was considered long enough to observe a population increase after a low phase, because the species studied here for possible recovery have life history characteristics typical to ‘fast’ species along the ‘slow‐fast’ continuum (Sæther 1987; Gaillard et al. 1989; Koons et al. 2014; see ‘Species Traits’ below). Moreover, total pair number of waterbirds breeding in eutrophic lakes increased consistently in Finland during late 1980s but decreased again in the early 1990s (Kauppinen and Väänänen 1999; Piha et al. 2025), meaning that a longer period would not have been suitable to study possible recovery from presumed population lows. Finally, long‐term population trends (SQ 5) were studied using species‐specific time series data combined from 11 communities for which continuous annual data were available (i.e., no missing years, see above) for 1987–2022 (Table S2). Information on the use of data from each of the 36 study lakes to answer the specific study questions is given in Table S2.

### 2.3 Species Traits

We used body mass (from Piha et al. 2018), clutch size (from Lehikoinen et al. 2011), and age at 1st reproduction (from Cramp and Simmons 1977, 1980) as species life history variables. Body mass could affect a species' tolerance (resistance) to cold weather, whereas clutch size and age at 1st reproduction could affect the rate at which populations of a species recover from a low phase. The long‐term average temperature experienced by individuals of a species over its range (species temperature index, STI) is a basic measure that has been used in studies addressing responses of birds and other animals to climate warming (Devictor et al. 2008). Gaget et al. (2020) calculated STIs for European wintering waterbirds as the average of the mean temperature in January across the nonbreeding species range in the African‐Eurasian region covered by the agreement on the conservation of African‐Eurasian migratory waterbirds (see the original article for further information). In addition to STIs, Gaget et al. (2020) calculated a thermal niche range for each species (species temperature range, STR) as the interval between the average temperatures of the thermal minimum (5% lower) and maximum (95% upper) of the nonbreeding species thermal range. We used the STI and STR values provided by Gaget et al. (2020) to measure species thermal affinity in the winter. We expected that species with lower STR (narrow temperature range, hence low thermal flexibility) should be more vulnerable to harsh wintering conditions. It is not straightforward to predict the association between STI and vulnerability to harsh wintering conditions because species with low STI (wintering in more northern areas) may be either more vulnerable (face colder winter weather) or less vulnerable (have adapted to cold wintering conditions) to harsh winters. Species‐specific values of the species traits are presented in Table S3.

### 2.4 Statistical Analyses

We used two approaches to test the impact of harsh wintering conditions on population dynamics of the species (see ‘Winter Severity Data’ and ‘Bird Population Data’ above). First, we used general linear mixed‐effects models to study the overall importance of wintering conditions in driving population dynamics of a species (SQ 1). All the time series meeting the data criteria (see ‘Bird Population Data’) were included in the same species‐specific analysis, with population change between years t and t‐1 (population growth rate; log_e_ *N*_*t*_—log_e_ *N*_*t*‐1_, where *N*_*t*_ is the number of breeding pairs in year *t* and *N*_*t*‐1_ is the number of breeding pairs in year *t*‐1) as the response variable, WSI, population density in year t‐1 (log_e_ *N*_*t*‐1_) and year as explanatory variables and lake ID as a random factor. Population density in year t‐1 was included to account for density dependence (Gunnarsson et al. 2013) and year was included to ensure that a common trend in the time series (i.e., both the response variable (population growth rate) and the explanatory variable in focus (WSI) showed a temporal trend) did not confound the effect of the explanatory variable in focus (Freckleton 2002; Lindström and Forchhammer 2010; Pöysä 2025). Second, we used general liner mixed‐effects models to study if population response to cold winters (resistance to exceptionally cold wintering conditions, these being represented by the five coldest winters) differs from that to mild winters (the five mildest winters) (SQ 2). Population growth rate was used as the response variable (N_*t*_ representing the number of breeding pairs in the year after a cold (or mild) winter and N_*t*‐1_ representing the number of breeding pairs in the year preceding the cold (or mild) winter), winter type as two‐level fixed factor (severe, mild; the latter level was represented by the intercept in the models), population density in year t‐1 (log_e_ *N*_*t*‐1_) as fixed covariate and lake ID as random factor. Distributional assumptions of linear mixed‐effects models were checked and plots of residuals versus predicted values suggested slight heteroscedasticity for some models. However, recent simulations have shown that linear mixed‐effects models, especially estimates of fixed effects, are robust even to severe violations of model assumptions (including homoscedasticity; Schielzeth et al. 2020; Knief and Forstmeier 2021). Hence, we believe that the slight violations of the homoscedasticity assumption do not bias the estimates and affect inferences concerning the fixed effect of interest (WSI). In one case (the model used to test for difference in growth rate of Great Crested Grebe ( *Podiceps cristatus* ) populations between exceptionally cold and exceptionally mild winters), a convergence problem appeared; results from this model are not reported. We used linear regression to study recovery rate of breeding numbers (population recovery) after the three consecutive exceptionally cold winters (SQ 3). Species‐specific regressions were calculated using standardized (mean = 0, variance = 1) annual pair numbers for the five consecutive years (1987–1991) as the dependent variable and year as the independent variable; slopes of the species‐specific regressions indicate the degree of recovery (hereafter, recovery rate). Similarly, we used slopes from linear regressions (based on standardized pair numbers; mean = 0, variance = 1) as indices of species‐specific long‐term (1987–2022) population trends (used to answer SQ 5).

We used univariate Kendall rank correlation to study if there is an association between population resistance to cold wintering conditions and species traits body mass, STR, and STI, as well as between population recovery rate and species traits age at 1st reproduction and clutch size (SQ 4). We used a rank correlation‐based approach instead of parametric correlation to avoid effects of extreme values on the results.

When reporting *p*‐values, we used the evidence‐based language and terminology recommended by Muff et al. (2022) instead of the traditional null‐hypothesis significance testing with a fixed cutoff value of *p* (Wasserstein et al. 2019). All statistical analyses were performed in SYSTAT 13.

## 3 Results

### 3.1 Responses to Varying Wintering Conditions

Even though WSI showed considerable between‐year variation, there was strong evidence that it decreased during the study period 1977–2022 (Figure 1; slope = −1.833, SE = 0.68, *F*_1,44_ = 7.252, *p* = 0.01).

Population growth rates of the species were generally not affected by variation in winter harshness, except in the Common Coot, for which there was strong evidence that population growth rate decreased with increasing winter harshness (Table 1; Table S4 and Figure S3). Nor did a closer comparison of population growth rates of individual species between years after the five coldest winters and years after the five mildest winters reveal a noticeable effect of exceptionally cold winters on population growth rates (Table 1; Table S5 and Figure S4). The Common Coot was a clear exception also in this analysis: there was very strong evidence that population growth rate was affected by winter type, being negative after the coldest winters when compared with that after the mildest winters (Table 1; Figure S4). In addition, there was weak evidence that the population growth rate of the Garganey (*Spatula querquedula*) was affected negatively by exceptionally cold winters compared with mild winters (Table 1).

**TABLE 1:** Effect of winter severity on population growth rate of waterbird species as predicted from general linear mixed‐effects models in which winter severity was used as continuous predictor of population growth rate in time series data (left side of the table) or as a two‐level fixed factor in data representing only exceptionally cold and exceptionally mild winters (right side of the table).

Species	Winter severity as overall driver of population growth rate (SQ 1)					Comparison of population growth rate between years after exceptionally cold winters and years after exceptionally mild winters (SQ 2)				
	Estimate	SE	*t*	*p*	*n*	Estimate	SE	*t*	*p*	*n*
Great Crested Grebe, *Podiceps cristatus (Pcr)*	−0.001	0.001	−1.419	0.157	210					
Red‐necked Grebe, *Podiceps grisegena (Pgr)*	0.000	0.001	−0.503	0.618	50	−0.055	0.106	−0.517	0.610	39
Hornrd Grebe, *Podiceps auritus (Pau)*	−0.001	0.001	−1.461	0.147	116	−0.087	0.106	−0.825	0.414	68
Whooper Swan, *Cygnus cygnus (Ccy)*	0.000	0.000	0.302	0.763	97	−0.001	0.046	−0.032	0.974	121
Eurasian Wigeon, *Mareca penelope (Mpe)*	0.000	0.001	0.700	0.485	338	0.106	0.087	1.218	0.225	168
Northern Pintail, *Anas acuta (Aac)*	0.000	0.001	0.430	0.668	106	−0.017	0.137	−0.124	0.902	61
Mallard, *Anas platyrhynchos (Apl)*	−0.001	0.000	−1.573	0.116	474	−0.078	0.070	−1.110	0.269	187
Common Teal, *Anas crecca (Acr)*	0.000	0.000	−0.134	0.893	461	0.044	0.082	0.539	0.590	180
Garganey, *Spatula quequedula (Squ)*	−0.001	0.001	−1.051	0.297	80	−0.209	0.121	−1.728	0.090	77
Northern Shoveler, *Spatula clypeata (Scl)*	0.001	0.001	1.063	0.289	215	0.067	0.109	0.619	0.538	93
Common Goldeneye, *Bucephala clangula (Bcl)*	0.000	0.000	−0.227	0.820	525	0.121	0.063	1.916	0.057	189
Goosander, *Mergus merganser (Mme)*						−0.090	0.127	−0.708	0.484	54
Tufted Duck, *Aythya fuligula (Afu)*	0.000	0.001	0.433	0.666	102	−0.061	0.122	−0.500	0.619	116
Common Pochard, *Aythya ferina (Afe)*	−0.001	0.001	−0.867	0.390	59	−0.088	0.130	−0.677	0.502	78
Common Coot, *Fulica atra (Fat)*	−0.002	0.001	−2.833	0.005	133	−0.593	0.132	−4.506	0.000	64

*Note:* In the models on the right side, ‘exceptionally mild’ winters were represented by intercept and ‘Estimate’ refers to the effect of exceptionally cold winters compared with exceptionally mild winters. Outputs for full models with all predictors are presented in Table S4 and Table S5, respectively. SQ 1 and SQ 2 refer to study questions 1 and 2, respectively (see Introduction).

### 3.2 Population Trajectories After Three Consecutive Cold Winters

**FIGURE 2:**
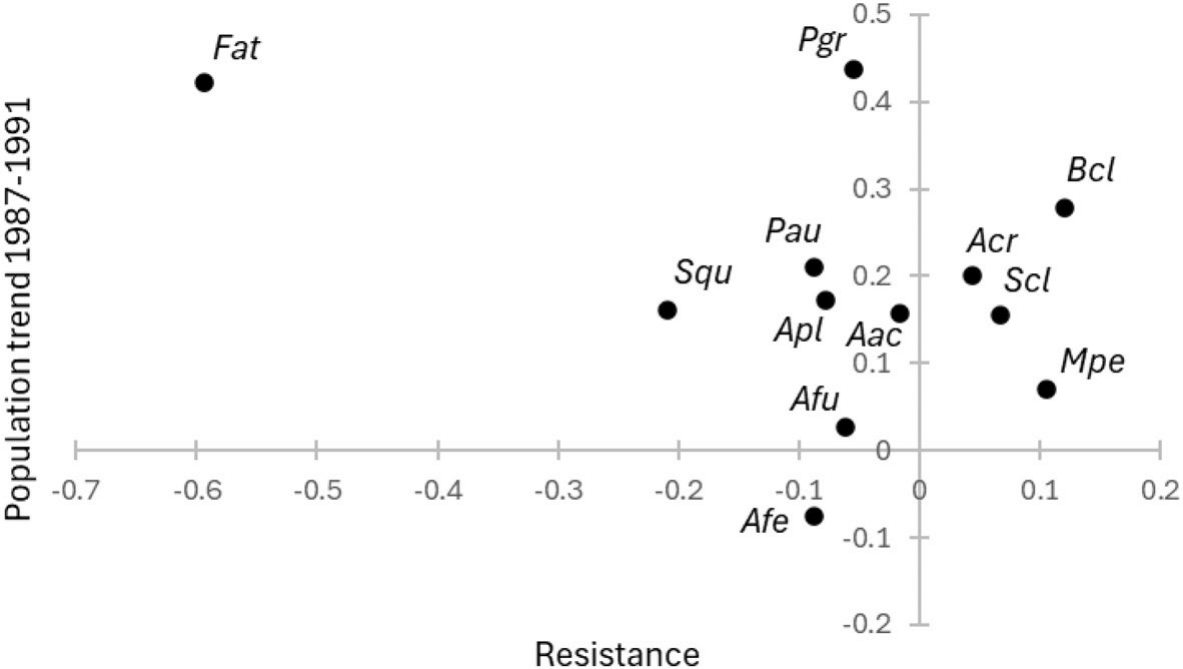
Population trend in 1987–1991 (slope from linear regression standardized number of breeding pairs vs. year, representing recovery rate) in relation to population resistance to cold winters (‘Estimate’ from the comparison of population growth rates between years after exceptionally cold winters and years after exceptionally mild winters, i.e., right side of Table 1) for waterbird species. See Table 1 for species abbreviations (note that, due to model convergence problems, resistance could not be estimated for Great Crested Grebe and data for the Whooper Swan and Goosander were not sufficient to calculate population trend in 1987–1991).

Species differed quite a lot with respect to population trajectories in 1987–1991, i.e., after the period of the three consecutive cold winters 1984/1985–1986/1987: slopes of the regressions of standardized pair numbers versus year ranged from −0.139 to 0.438 (see Figure 2; evidence of recovery (positive slope) very strong for Common Coot (*Fat*) and Common Goldeneye (*Bcl*), strong for Common Teal ( *Anas crecca* , *Acr*) and moderate for Mallard ( *Anas platyrhynchos* , *Apl*) Red‐necked Grebe ( *Podiceps grisegena* , *Pgr*) and Northern Shoveler (*Spatula clypeata*, *Scl*); Table S6). Assuming these trajectories reflect recovery from the presumed population lows due to the three consecutive cold winters and that winter severity is an important driver of population dynamics in waterbirds, we could expect that species with low resistance to cold winters would show relatively more positive population trajectories (high recovery rate), whereas population trajectories of species with high resistance would not indicate consistent recovery during 1987–1991. While many species showed a positive population trend during 1987–1991, the Common Coot stands out by showing both low resistance and high recovery rate (Figure 2).

### 3.3 Population Resilience and Species Traits

Although responses to variation in winter severity were generally weak, notable among‐species variation occurred in terms of both resistance and recovery rate (see above and Figure 2). However, neither resistance nor recovery rate was associated with the species traits studied (Table 2).

**TABLE 2:** Kendall rank correlations between population resistance to cold wintering conditions and species traits body mass, STR and STI as well as between population recovery rate and species traits age at 1st reproduction and clutch size.

		Kendall rank correlation, τ	*p*	*n*
Resistance vs.	Body mass	−0.099	0.661	14
	STI	0.011	1.000	14
	STR	0.165	0.443	14
Recovery rate vs.	Age at 1st reproduction	0.376	0.132	13
	Clutch size	−0.144	0.540	13

*Note:* Population resistance to cold winters was measured as the effect from the comparison of population growth rates between years after exceptionally cold winters and years after exceptionally mild winters (i.e., ‘Estimate’ from right side of Table 1) and recovery rate as the slope from linear regressions of standardized number of breeding pairs vs. year in 1987–1991 (note that, due to model convergence problems, resistance could not be estimated for Great Crested Grebe and data for the Whooper Swan and Goosander were not sufficient to calculate population trend (recovery rate) in 1987–1991).

### 3.4 Population Resistance and Long‐Term Population Trends

**FIGURE 3:**
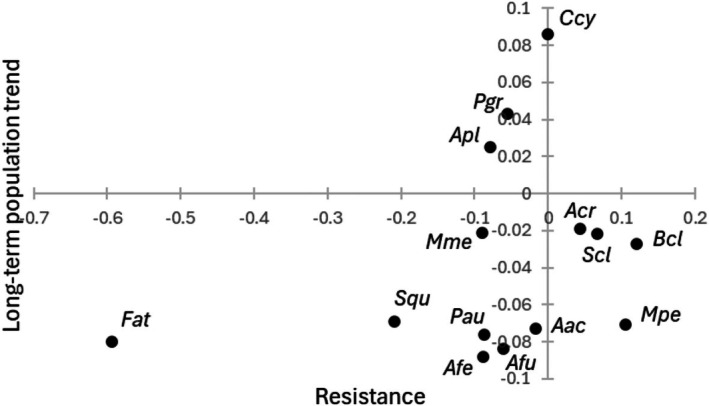
Long‐term (1987–2022) population trend (slope from linear regression standardized number of breeding pairs vs. year) in relation to population resistance to cold winters (‘Estimate’ from the comparison of population growth rates between years after exceptionally cold winters and years after exceptionally mild winters, i.e., right side of Table 1) for waterbird species. See Table 1 for species abbreviations.

While both negative and positive long‐term population trends were found (Table S7), there was no evidence of association between resistance and species‐specific long‐term population trend (Figure 3; Kendall rank correlation, τ = 0.231, *p* = 0.274, *n* = 14).

## 4 Discussion

We studied the resilience of waterbirds breeding in European boreal lakes to harsh weather conditions in the wintering areas, aiming to assess the possibility that climate warming could smooth waterbird population declines caused by other anthropogenic pressures. We found that, in general, wintering conditions had only a marginal impact on the dynamics (growth rate) of breeding populations of waterbirds. A notable exception was the Common Coot, in which cold winters affected the population growth rate negatively. A closer comparison of population growth rates of individual species between years of the five coldest winters and years of the five mildest winters confirmed the overall pattern: cold winters did not negatively affect population growth rates, except in the Common Coot and, less strongly so, in the Garganey. Hence, our results suggest that, excluding the Common Coot, populations of waterbirds breeding in European boreal lakes have a relatively strong resistance to cold weather conditions in their wintering areas. Furthermore, we found that, even though the population growth rate of most species did not show sensitivity to cold wintering conditions, population trajectories of six species showed at least moderate evidence of population increase after the period of three consecutive cold winters, suggesting a high recovery rate. Finally, we found little evidence of association between the resilience of breeding populations of waterbirds to harsh weather conditions in their wintering areas and species life history traits or species thermal niches in their wintering ranges, nor did we find evidence of association between resistance to cold winters and long‐term population trends.

### 4.1 Winter Severity and Waterbird Population Dynamics

Our findings are generally in line with Kauppinen and Väänänen (1999), who found little evidence of winter weather effect on breeding numbers of waterfowl in central Finland, but differ from those of Pavón‐Jordán et al. (2017), who reported that combined breeding numbers of waterbirds in Finland were positively associated with the NAO index in the previous winter, positive NAO indicating above‐average temperatures and precipitations in the northwestern wintering areas of waterbirds in Europe. The latter authors reported further that the association between combined waterbird numbers and NAO index was more positive for waterbirds breeding in eutrophic lakes, although no species‐specific results were presented. In our study, the Common Coot was the only species that showed both low resistance to harsh wintering conditions and high recovery rate after exceptionally cold winters. Common Coot is strongly associated with eutrophic lakes (Kauppinen 1993; Elmberg et al. 2020), and its numbers typically made up to 30% of the total number of pairs of all species in waterbird communities of eutrophic lakes in southern Finland (Lammi 1978; Pöysä 1984; Pöysä and Linkola 2021) before they started to decrease in the early 1990s (Piha et al. 2025). Hence, breeding numbers of the Common Coot probably contributed strongly to the combined breeding numbers of waterbirds in eutrophic lakes and, thus, affected the results of Pavón‐Jordán et al. (2017). Our findings underline the need to consider species‐specific responses to various ecological disturbances, including climate change. Analyses based on data pooled over species may lead to generalizations that do not apply to all species, not even in cases in which the species group represents an ecologically seemingly homogenous community occurring in the same habitat type such as waterbirds living in wetlands.

Why does the Common Coot differ from the other species with respect to resistance to cold winters? One reason for the difference probably is that Common Coots are relatively sedentary in the wintering areas compared to many other waterbird species. Indeed, ringing recovery data from the wintering areas suggest that the Common Coot does not undergo large‐scale cold‐weather movements in response to severe conditions, such movements being found e.g., for Eurasian Wigeon (*Mareca penelope*), Common Teal, Northern Pintail ( *Anas acuta* ), Northern Shoveler, Tufted Duck ( *Aythya fuligula* ), and Common Pochard ( *Aythya ferina* ) among the species studied here (Ridgill and Fox 1990). This behavior, and strong dependence on shallow ice‐free water in feeding, obviously makes the Common Coot highly vulnerable to impacts of cold winters, increasing mortality (Visser 1978; Cavé and Visser 1985). Our finding that harsh winters affect negatively Common Coot breeding numbers in European boreal lakes is consistent with findings from more southern Europe where Common Coots are resident. Cavé and Visser (1985) demonstrated for a local breeding population of Common Coot in the Netherlands that variation in annual survival rate, caused by winter severity, contributed to variation in annual numbers of breeding birds.

Garganey was another species in which population growth rate was negatively associated with cold winters. However, because the Garganey is a long‐distance migrant, wintering in Sub‐Saharan Africa (Scott and Rose 1996), it is unlikely that wintering conditions in central‐western Europe directly affect its breeding numbers in northern Europe. Rather, we suggest that the result is explained by temperature‐driven variation in the migratory behavior of the species. It has been found that high spring (mid‐April—late May) temperature, probably stimulating prolonged (northern) spring migration, results in higher breeding numbers of Garganey in Finland (Pöysä and Väänänen 2014), low spring temperatures causing an opposite effect. Similarly, Kjeldsen (2008) found a positive correlation between the average temperature in March and the breeding numbers of Garganey in Vejlerne, Denmark. Hence, low temperatures in winter and early spring along the migration route of the species in Europe probably explain why the population growth rate of the species was lower after severe winters.

### 4.2 Species Traits and Population Resilience

As to the variables indicating winter thermal affinity, STI and STR, our findings suggest these probably are too crude measures to indicate species‐specific ability to cope with varying climatic conditions in the wintering areas. STI and STR measure thermal conditions (average and range, respectively) in the winter distribution range of a species, whereas mobility may be a more important trait to measure vulnerability of a species to impacts of harsh wintering conditions (Ridgill and Fox 1990; see the Common Coot example above). Similarly, Dalby et al. (2013) concluded that temperature via thermoregulatory costs is less important than for example, feeding ecology in shaping mid‐winter distribution of dabbling ducks in Western Europe. Considering other species traits than those analyzed in this study, the scatter of species‐specific data points along the resistance and recovery rate gradients (Figure 2) does not suggest consistent patterns with respect to any of the factors suggested to be important drivers of waterbird population declines in Finland. First, considering breeding habitat affinity (habitat deterioration probably being a more serious problem to species preferring eutrophic lakes; Pöysä and Linkola 2021; Holopainen et al. 2024), Horned Grebe (*Podiceps auritus, Pau*), Northern Shoveler (*Scl*), Common Pochard (*Afe*) and Common Coot (*Fat*) are associated with eutrophic waters (Kauppinen 1993; Elmberg et al. 2020; see also Lehikoinen et al. 2016; Pöysä et al. 2023) but differ clearly in terms of both resistance and recovery rate, a similar discrepancy applying to two habitat generalists, the Common Goldeneye (*Bcl*) and Tufted Duck (*Afu*). Second, considering foraging ecology (eutrophication‐caused changes in habitat quality probably being foraging guild specific; Pöysä et al. 2023; Holopainen et al. 2024), Great Crested Grebe (*Pcr*) and Red‐necked Grebe (*Pgr*) are pursuit‐feeding piscivores (Pöysä 1983; Pöysä et al. 2023) but represent the opposite extremes in terms of recovery rate. Common Goldeneye and Tufted Duck in turn are bottom‐feeding diving ducks (Pöysä 1983; Pöysä et al. 2023) but differ in terms of both resistance and recovery rate. All in all, species showing similarity in terms of resistance (values between −0.1 and 0 on the horizontal axis in Figure 2) represent three different foraging guilds (pursuit‐feeding piscivores, bottom‐feeding diving ducks, surface‐feeding dabbling ducks). Third, considering nest sites that make species differently vulnerable to predation risk (Pöysä et al. 2023; Holopainen et al. 2024), among the three species with highest recovery rate, the Red‐necked Grebe and Common Coot are wetland nesters, whereas Common Goldeneye is cavity‐nester, wetland nesting and cavity nesting representing opposite extremes in terms of vulnerability to predation risk by two alien predators, the Raccoon Dog ( *Nyctereutes procyonoides* ) and American Mink ( *Neovison vison* ) (Pöysä et al. 2023). Moreover, Common Coot, Horned Grebe, Common Pochard and Great Crested Grebe belong to the same nest site guild and are similarly vulnerable to the two alien predators (wetland nesters; Pöysä et al. 2023; Holopainen et al. 2024); however, they differed clearly in recovery rate. Hence, it seems unlikely that nest predation could explain among‐species differences (or similarities) in recovery rate.

Even though the species that showed high recovery rate were relatively resistant to cold winters and were probably not recovering from a cold winter‐caused population low (except the Common Coot), positive population trajectories during the period of five consecutive relatively warm winters suggest that climate change‐caused overall warming of winters at least does not affect negatively populations recovering from low phases. Negative effects could arise, for example, if winter warming‐caused shifts in the distribution of species (Lehikoinen et al. 2013; Pavón‐Jordán et al. 2019) would result in mismatches between new wintering areas and the network of currently protected areas (Guillemain and Hearn 2017; Marchowski et al. 2020; Pavón‐Jordán et al. 2020). Interestingly, high recovery rate appeared not to depend on clutch size or age at 1st reproduction, although the number of species (sample size) in the analysis was relatively small and further studies are needed to confirm the result. Even though this result should be considered preliminary, it is good news for conservation programmes aimed to increase breeding numbers of declining species whatever the position of the species on the continuum of these life history traits.

### 4.3 Potential Limitations of the Study

There are other potential limitations in our study that need attention. First, related to the case of Garganey (see above), one might suggest that the WSI calculated for central‐western Europe may not be representative for species wintering in more southern areas. However, we consider the WSI generally applicable for species wintering in Europe. WSI is based on temperature which is typically correlated across wide geographical areas. For example, the WSI calculated for central‐western Europe (used in the analyses) in 1977–2022 was correlated with WSI calculated for western‐southern Europe (see ‘Materials and Methods’, ‘Winter Severity Data’). Hence, if a winter is cold (or mild) in central‐western Europe it is cold (or mild) also in more southern wintering areas of waterbirds in Europe, although in general winters are milder in southern Europe than in northern Europe (see Figure 1 and Figure S2 for comparison of the annual WSI values in central‐western Europe versus western‐southern Europe). Consequently, in the analyses, the WSI of central‐western Europe should statistically reveal an effect of winter severity also on population dynamics of species wintering in more southern areas if such an effect exists, although the numerical value of the effect estimate would differ due to the difference in the level of winter severity between south and north. All in all, because winters typically are much milder in southern Europe than in northern Europe, we consider it unlikely that the population growth rate of waterbirds wintering in southern Europe would be affected negatively by winter severity, a general pattern that emerged from the analyses. Related to the potential issue concerning differences in winter distribution of the species with respect to WSI, one might question the assumption that birds breeding in any of the local communities in the southern part of Finland are affected similarly by weather conditions in the wintering areas. We consider this assumption biologically realistic, because weather conditions in the wintering areas in central‐western or southern Europe should affect individual birds similarly wherever they breed in southern Finland.

Second, sample size in the analyses varied considerably among the species studied (resistance: *n* = 39–525, Table 1; recovery rate: *n* = 10–85, Table S6), and one might suggest that tests for some species did not return significant effects simply because of low sample size. However, whether a test showed statistically strong support for an effect or not clearly was not a consequence of sample size. For example, the three species with the highest sample sizes (the Mallard, Common Teal, and Common Goldeneye) did not show any evidence of the impact of winter severity on population growth rate, whereas the species with one of the lowest sample sizes (the Common Coot) showed strong or very strong evidence, depending on the SQ, of negative effect of cold winters on population growth rate (Table 1). Similarly, two species with the lowest sample sizes (the Red‐necked Grebe and Common Coot) showed moderate or very strong evidence of recovery (Table S6). Hence, low power of the statistical tests due to small sample size appears not to be an issue with the species‐specific results reported here.

Third, one might ask if the 36 eutrophic lakes studied here are a representative sample of the breeding lakes of waterbirds in the region. While a larger number of lakes from different parts of Finland, of course, would have been helpful, we believe that the geographical distribution of the study lakes makes the sample representative enough (see ‘Bird Population Data’ and Figure S2 and Table S2 for lake coordinates; see also Pöysä and Linkola 2021; Pöysä et al. 2023). Furthermore, species‐specific long‐term population trends in our data were correlated with corresponding long‐term trends in the Finnish national waterbird monitoring data covering the whole country (Table S7; see Piha et al. 2025 for further information concerning the monitoring data), supporting the view that the data analyzed here are representative of larger geographical areas.

Finally, our study was based on population level count data in which possible impacts of winter severity on fecundity and mortality of individuals were assumed to be reflected but appeared not to be realized in terms of variation in population growth rate. To confirm findings of this type of correlative analysis, we urgently need demographic studies coupled with tracking the performance of individual birds to reveal impacts of wintering conditions, or lack of them, on critical demographic traits (e.g., Trinder et al. 2009; Aðalsteinsson et al. 2025; Kujala et al. 2025), extending to impacts on breeding numbers (e.g., Piironen et al. 2025).

### 4.4 Implications With Respect to Global Climate Change

All in all, our findings have important implications when it comes to projected climate warming‐induced changes in the wintering conditions of migratory waterbirds and probably other avian species in northern Europe. First, considering short‐term changes, persistence of warm winter weather in northwestern Europe has increased in recent decades (Spanjers et al. 2025) and the frequency of ice‐free winters is predicted to increase in the coming few decades (Sharma et al. 2019). Our results suggest that most waterbird species will not greatly benefit from the warming trend, because their breeding populations appeared to be relatively resistant to harsh wintering conditions, the Common Coot being a notable exception. Hence, our results do not support the suggestion by Pavón‐Jordán et al. (2017) that global warming could generally benefit waterbirds by increased winter survival due to more favorable winter weather conditions. The bad news thus is that warmer winters may not provide a mechanism that could mitigate negative impacts of other anthropogenic stressors on breeding populations of waterbirds in northern Europe. Increased predation pressure due to the two alien predators (see above) and over‐eutrophication and associated environmental changes have been identified as the main reasons for the decline of waterbird breeding numbers in boreal Europe (Lehikoinen et al. 2017; Pöysä and Linkola 2021; Pöysä et al. 2023; Holopainen et al. 2024), these probably overriding any positive effects of milder winters on breeding populations. This concerns especially the Common Coot, the breeding numbers of which are strongly declining (Piha et al. 2025; see also Table S7) even though the species should benefit from warming winters, as found in this study. Second, climate warming‐induced changes in global climate regulators may have unexpected impacts on weather conditions in Europe over a longer term. The Atlantic meridional overturning circulation (AMOC), transporting heat from the Southern Hemisphere to the Northern Hemisphere, is projected to weaken due to climate warming (e.g., Weijer et al. 2020), some models predicting a collapse even well before 2100 (Ditlevsen and Ditlevsen 2023). While there is considerable uncertainty on the tipping point of AMOC (Ditlevsen and Ditlevsen 2023; Zimmerman et al. 2025), a strongly reduced AMOC may decrease winter temperatures dramatically over northwestern Europe (van Westen and Baatsen 2025). Considering this projected long‐term change in the wintering conditions in central‐western Europe, our finding that most of the waterbird species studied here are relatively resistant to cold winters is good news as these species should largely resist the predicted cooling.

## Author Contributions

**Hannu Pöysä:** conceptualization (lead), data curation (equal), formal analysis (lead), investigation (lead), visualization (lead), writing – original draft (lead), writing – review and editing (equal). **Esa Lammi:** data curation (equal), investigation (equal), writing – review and editing (equal). **Veli‐Matti Väänänen:** data curation (equal), investigation (equal), writing – review and editing (equal).

## Funding

The authors have nothing to report.

## Conflicts of Interest

The authors declare no conflicts of interest.

## Supporting information


**Data S1:** ece373718‐sup‐0001‐Supinfo1.xlsx.

**Figure S1:** Location of the 36 study lakes in the southern part of Finland and six weather stations (blue asterisks) in central‐western Europe. Exact locations of the study lakes are given in the ETRS‐TM35FIN coordinate system in the right‐hand panel. Distance between the tick marks on the x (E coordinates) and y (*N* coordinates) axes equals 50 km in the field. Names and coordinates of the weather stations are given in Table S1 (central‐western Europe) and study lakes in Table S2.
**Figure S2:**. Mean of the winter severity index (WSI) of six weather stations in western‐southern Europe in 1977–2022. The weather stations are given in Table S1 (western‐southern Europe). The sum of mean daily temperatures that were below zero between 1 December and 28 February was first calculated for each station and the mean of the station‐specific values was then calculated (absolute values used; see Materials and Methods in the main article). See Figure 1 in the main text for the corresponding WSI for six weather stations in central‐western Europe.
**Figure S3:**. Effect of winter severity (WSI) on population growth rate for different waterbird species. Figures are drawn based on predicted values from species‐specific models presented in Table S4. Trend lines (dashed lines) are drawn for illustrative purposes (see Table S4 for statistical evidence of the dependence of growth rate of WSI and the main text for further details).
**Figure S4:**. Population growth rates in years after exceptionally cold winters (winter type 1) and in years after exceptionally mild winters (winter type 2). Figures are drawn based on predicted values from species‐specific models presented in Table S5.
**Table S1:**. Weather stations used to calculate winter severity indices (WSI) for main wintering areas of waterbirds breeding in Finland (see Material and Methods in the main article). Names, numbers and coordinates of the stations are according to the source: http://www.ecad.eu (Klein Tank et al. 2002).
**Table S2:**. Name, ID and coordinates of the studied lakes (communities) as well as the first year, last year, length, number of missing years (i.e., no bird censuses done) and number of species (i.e., species that provided data to answer at least one of the SQs) for each lake‐specific time series. The suitability of the lake‐specific time series data to answer a particular study question is also specified (x) as well as the data source (the most recent article in which a dataset was used is given, together with the original source (if not our own data); see also the main text). Note that lake IDs used here differ from those used in Pöysä and Linkola (2021, Supporting Information Table A1).
**Table S3:**. Species traits used in analyses.
**Table S4:**. General linear mixed‐effects models for the overall importance of wintering conditions in driving population dynamics (growth rate) of different waterbird species. Population growth rate was the response variable and severity of the previous winter (WSI), Year and population density in year t‐1 (Density) were used as explanatory variables. Lake ID was included as a random factor in all models.
**Table S5:**. General linear mixed‐effects models for comparing population growth rates between years after exceptionally cold winters and years after exceptionally mild winters. Winter type ‘mild’ was included in the intercept; ‘Estimate’ for variable ‘WSI’ is exceptionally cold winters compared with exceptionally mild winters.
**Table S6:**. Linear regressions between standardized annual pair number and year (time) in 1987–1991 for different waterbird species to examine population recovery after three consecutive exceptionally cold winters.
**Table S7:**. Long‐term population trends (Slope) for different waterbird species as indicated by slopes of linear regressions between standardized annual pair number and year in 1987–2022 (*n* = 36 in all cases). Species' long‐term population trends (percentage change of breeding numbers during 1986–2025, last column) based on the Finnish national monitoring data are also given (from Piha et al. 2025). Species' population trend indices are strongly correlated between the two data sets (Slope vs. Percentage population change, Kendall rank correlation, τ = 0.692, *p* = 0.0007, *n* = 14).

## Data Availability

All the required data are uploaded as Supporting Information.
