# Supplementary material for: Resilience of Breeding Boreal Waterbirds to Harsh Wintering Conditions: Could Climate Warming Smooth Population Declines?
Source: Ecol Evol. 2026 May 27;16(6):e73718. doi: 10.1002/ece3.73718 (PMC13240112; doi:10.1002/ece3.73718)
Supplement: Supplementary file 2 — Figure S1: Location of the 36 study lakes in the southern part of Finland and six weather stations (blue asterisks) in central‐western Europe. Exact locations of the study lakes are given in the ETRS‐TM35FIN coordinate system in the right‐hand panel. Distance between the tick marks on the x (E coordinates) and y (N coordinates) axes equals 50 km in the field. Names and coordinates of the weather stations are given in Table S1 (central‐western Europe) and study lakes in Table S2. Figure S2:. Mean of the winter severity index (WSI) of six weather stations in western‐southern Europe in 1977–2022. The weather stations are given in Table S1 (western‐southern Europe). The sum of mean daily temperatures that were below zero between 1 December and 28 February was first calculated for each station and the mean of the station‐specific values was then calculated (absolute values used; see Materials and Methods in the main article). See Figure 1 in the main text for the corresponding WSI for six weather stations in central‐western Europe. Figure S3:. Effect of winter severity (WSI) on population growth rate for different waterbird species. Figures are drawn based on predicted values from species‐specific models presented in Table S4. Trend lines (dashed lines) are drawn for illustrative purposes (see Table S4 for statistical evidence of the dependence of growth rate of WSI and the main text for further details). Figure S4:. Population growth rates in years after exceptionally cold winters (winter type 1) and in years after exceptionally mild winters (winter type 2). Figures are drawn based on predicted values from species‐specific models presented in Table S5. Table S1:. Weather stations used to calculate winter severity indices (WSI) for main wintering areas of waterbirds breeding in Finland (see Material and Methods in the main article). Names, numbers and coordinates of the stations are according to the source: http://www.ecad.eu (Klein Tank et al. 2002). Table S2:. Name, ID [file ECE3-16-e73718-s001.docx]

Supporting Information to:

**Resilience of Breeding Boreal Waterbirds to Harsh Wintering Conditions: Could Climate Warming Smooth Population Declines?**

Hannu Pöysä, Esa Lammi, Veli-Matti Väänänen

**Table S1**. Weather stations used to calculate winter severity indices (WSI) for main wintering areas of waterbirds breeding in Finland (see Material and Methods in the main article). Names, numbers and coordinates of the stations are according to the source: http://www.ecad.eu (Klein Tank et al. 2002)

|  |  |  |  |
| --- | --- | --- | --- |
| **Central-western Europe** | **Number** | **Latitude** | **Longitude** |
| Malmo | 5175 | 55:36:36 | 013:04:48 |
| Groningen | 163 | 53:13:00 | 006:33:00 |
| Berlin-Dahlem | 41 | 52:27:50 | 013:18:06 |
| Strasbourg-Entzheim | 323 | 48:32:57 | 007:38:25 |
| Munchen | 52 | 48:09:51 | 011:32:39 |
| Zuerich/Fluntern | 244 | 47:22:59 | 008:34:00 |
|  |  |  |  |
| **Western-southern Europe** |  |  |  |
| Cet Central England | 257 | 52:25:12 | -001:49:48 |
| Oxford | 274 | 51:46:00 | -001:16:00 |
| Caen-Carpiquet | 738 | 49:10:48 | -000:27:21 |
| Rennes-St Jacques | 322 | 48:04:08 | 001:44:02 |
| Bordeaux-Merignal | 34 | 44:49:50 | -000:41:29 |
| Toulouse-Beagnal | 33 | 43:37:15 | 001:22:44 |


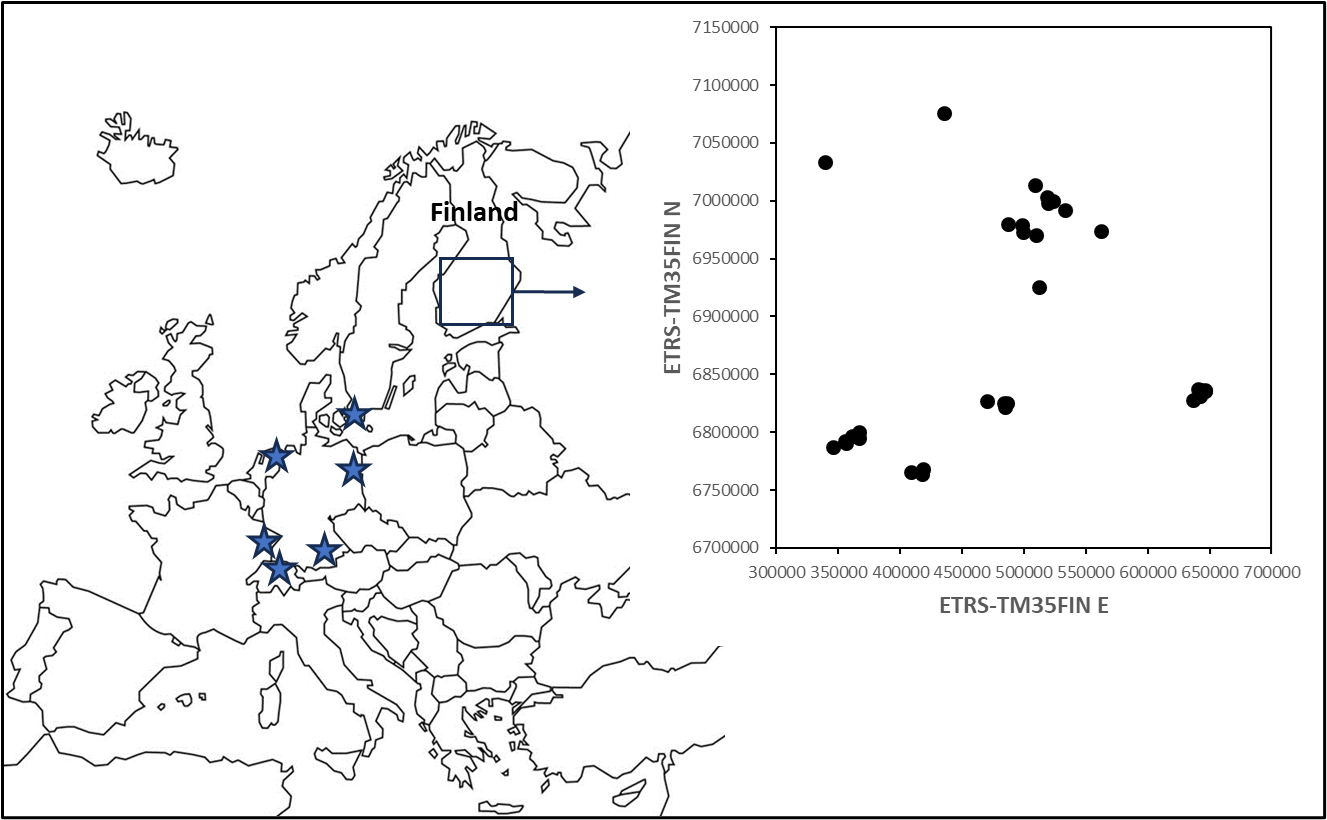


**Fig. S1.** Location of the 36 study lakes in the southern part of Finland and six weather stations (blue asterisks) in central-western Europe. Exact locations of the study lakes are given in the ETRS-TM35FIN coordinate system in the right-hand panel. Distance between the tick marks on the x (E coordinates) and y (N coordinates) axes equals 50 km in the field. Names and coordinates of the weather stations are given in Table S1 (central-western Europe) and study lakes in Table S2.


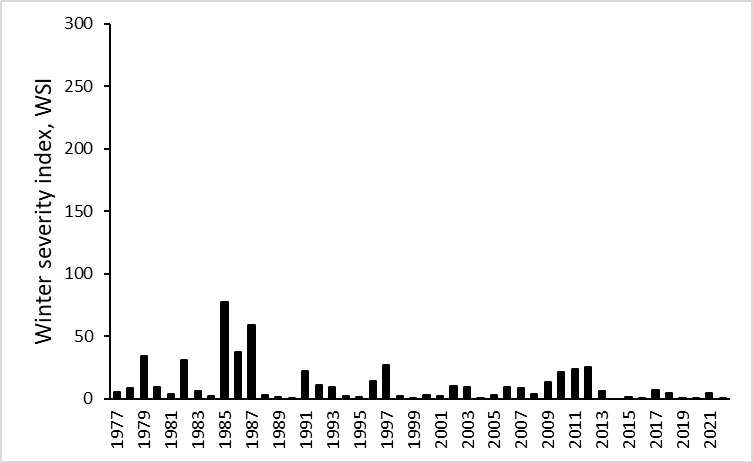


**Fig. S2**. Mean of the winter severity index (WSI) of six weather stations in western-southern Europe in 1977–2022. The weather stations are given in Table S1 (western-southern Europe). The sum of mean daily temperatures that were below zero between 1 December and 28 February was first calculated for each station and the mean of the station-specific values was then calculated (absolute values used; see Materials and Methods in the main article). See Fig. 1 in the main text for the corresponding WSI for six weather stations in central-western Europe.

**Table S2**. Name, ID and coordinates of the studied lakes (communities) as well as the first year, last year, length, number of missing years (i.e. no bird censuses done) and number of species (i.e. species that provided data to answer at least one of the SQs) for each lake-specific time series. The suitability of the lake-specific time series data to answer a particular study question is also specified (x) as well as the data source (the most recent article in which a dataset was used is given, together with the original source (if not our own data); see also the main text). Note that lake IDs used here differ from those used in Pöysä and Linkola (2021, Supplementary material Table A1).

|  |  |  |  |  |  |  |  |  | **Data suitable to answer different study questions (SQ)** | | | |  |
| --- | --- | --- | --- | --- | --- | --- | --- | --- | --- | --- | --- | --- | --- |
| **Lake name** | **Lake ID** | **Latitude, N** | **Longitude, E** | **First year** | **Last year** | **Length** | **Missing years** | **Species** | **SQ 1** | **SQ 2** | **SQ 3** | **SQ 5** | **Source** |
|  |  |  |  |  |  |  |  |  |  |  |  |  |  |
| **Kutajärvi** | 1 | *61° 2,216'* | *25° 29,294'* | 1977 | 2022 | 46 | 0 | 13 | x | x | x | x | Pöysä et al., 2023 |
| **Sairakkalanjärvi** | 2 | *61° 0,688'* | *25° 18,842'* | 1987 | 2022 | 36 | 0 | 12 | x | x | x | x | Pöysä et al., 2023 |
| **Mustajärvi** | 3 | *61° 0,157’* | *25° 28,302'* | 1987 | 2022 | 36 | 0 | 7 | x | x | x | x | Pöysä et al., 2023 |
| **Siikalahti** | 4 | *61° 33,593'* | *29° 34,100'* | 1980 | 1986 | 7 | 0 | 14 | x | x | x |  | Pöysä et al., 2023 |
| **Kuvalahti** | 5 | *61° 37,406'* | *29° 45,497'* | 1991 | 2022 | 32 | 0 | 8 | x | x | x |  | Pöysä et al., 2023 |
| **Kivilahti** | 6 | *61° 38,139'* | *29° 45,775'* | 1991 | 2022 | 32 | 0 | 15 | x | x | x |  | Pöysä et al., 2023 |
| **Pyvältölampi** | 7 | *61° 38,024'* | *29° 42,170'* | 1991 | 2022 | 32 | 0 | 10 | x | x | x |  | Pöysä et al., 2023 |
| **Humallampi** | 8 | *61° 35,436'* | *29° 40,604'* | 1991 | 2022 | 32 | 0 | 10 | x | x | x |  | Pöysä et al., 2023 |
| **Putrolampi** | 9 | *61° 38,204'* | *29° 40,268'* | 1991 | 2022 | 32 | 0 | 8 | x | x | x |  | Pöysä et al., 2023 |
| **Tönkilampi** | 10 | *61° 38,634'* | *29° 39,436'* | 1991 | 2022 | 32 | 0 | 9 | x | x | x |  | Pöysä et al., 2023 |
| **Valkialampi** | 11 | *61° 37,784'* | *29° 40,528'* | 1991 | 2022 | 32 | 0 | 8 | x | x | x |  | Pöysä et al., 2023 |
| **Räyringin järvi** | 12 | *63° 23,453'* | *23° 47,425'* | 1977 | 1982 | 6 | 0 | 10 |  | x |  |  | Pöysä et al., 2023; Tossavainen and Tossavainen, 1984 |
| **Nurmesjärvi** | 13 | *63° 48,380'* | *25° 41,638'* | 1981 | 1985 | 5 | 0 | 14 |  | x |  |  | Pöysä et al., 2023; Virta, 1985 |
| **Ritvalan Vähäjärvi** | 14 | *61⁰11,393'* | *24⁰08,177'* | 1998 | 2015 | 18 | 1 | 14 |  | x |  |  | Pöysä and Linkola, 2021 |
| **Hiiramenjärvi** | 15 | *61⁰16,989'* | *24⁰30,666'* | 1998 | 2015 | 18 | 6 | 9 |  | x |  |  | Pöysä and Linkola, 2021 |
| **Jouttijärvi** | 16 | *61⁰14,328'* | *24⁰18,056'* | 1998 | 2015 | 18 | 2 | 10 |  | x |  |  | Pöysä and Linkola, 2021 |
| **Särkijärvi** | 17 | *61⁰16,628'* | *24⁰24,677'* | 1999 | 2014 | 16 | 1 | 4 | x | x |  |  | Pöysä and Linkola, 2021 |
| **Urkanjärvi** | 18 | *61⁰18,638'* | *24⁰30,927'* | 1998 | 2012 | 15 | 5 | 6 |  | x |  |  | Pöysä and Linkola, 2021 |
| **Valkjärvi** | 19 | *61⁰16,759'* | *24⁰29,553'* | 1998 | 2015 | 18 | 1 | 8 |  | x |  |  | Pöysä and Linkola, 2021 |
| **Vekurinjärvi** | 20 | *61⁰13,358'* | *24⁰19,903'* | 1998 | 2015 | 18 | 2 | 9 |  | x |  |  | Pöysä and Linkola, 2021 |
| **Keskimmäinen** | 21 | *62° 53,277'* | *28° 14,036'* | 1977 | 2009 | 33 | 9 | 13 | x | x | x |  | Pöysä et al., 2023; Kauppinen and Väänänen, 1999 |
| **Tuomiojärvi** | 22 | *62° 27,646'* | *27° 14,678'* | 1977 | 2009 | 33 | 7 | 13 | x | x | x |  | Pöysä et al., 2023; Kauppinen and Väänänen, 1999 |
| **Pitkäjärvi** | 23 | *62° 51,971'* | *27° 11,378'* | 1977 | 2009 | 33 | 7 | 13 | x | x | x |  | Pöysä et al., 2023; Kauppinen and Väänänen, 1999 |
| **Apaja-Kumpunen** | 24 | *63° 3,119'* | *27° 39,260'* | 1987 | 2009 | 23 | 1 | 10 | x | x | x |  | Pöysä et al., 2023; Kauppinen and Väänänen, 1999 |
| **Pohjalampi** | 25 | *62° 56,995'* | *26° 44,868'* | 1985 | 2009 | 25 | 1 | 12 | x | x | x |  | Pöysä et al., 2023; Kauppinen and Väänänen, 1999 |
| **Pörönlampi** | 26 | *62° 53,379'* | *26° 59,295'* | 1986 | 2009 | 24 | 0 | 11 | x | x | x |  | Pöysä et al., 2023; Kauppinen and Väänänen, 1999 |
| **Suojärvi** | 27 | *62° 56,551’* | *26° 58,400'* | 1984 | 2009 | 26 | 10 | 11 |  | x |  |  | Pöysä et al., 2023; Kauppinen and Väänänen, 1999 |
| **Nironlahti** | 28 | *61° 33,584'* | *26° 41,893'* | 1986 | 2022 | 37 | 37 | 11 | x | x | x | x | Pöysä et al., 2023 |
| **Särkijärvi-Mäntyharju** | 29 | *61° 31,793'* | *26° 42,630'* | 1986 | 2022 | 37 | 37 | 10 | x | x | x | x | Pöysä et al., 2023 |
| **Viitalampi** | 30 | *61° 34,563'* | *26° 26,236'* | 1990 | 2022 | 33 | 33 | 13 | x | x | x |  | Pöysä et al., 2023 |
| **Tervajärvi** | 31 | *61° 33,783'* | *26° 44,929'* | 1986 | 2022 | 37 | 37 | 9 | x | x | x | x | Pöysä et al., 2023 |
| **Lapinjärvet** | 32 | *63° 7,539'* | *27° 25,439'* | 1985 | 2022 | 38 | 38 | 12 | x | x | x | x | Pöysä et al., 2023 |
| **Valkeinen** | 33 | *63° 6,457'* | *27° 23,472'* | 1985 | 2022 | 38 | 38 | 14 | x | x | x | x | Pöysä et al., 2023 |
| **Keskimmäinen** | 34 | *63° 8,079'* | *27° 29,753'* | 1985 | 2022 | 38 | 38 | 13 | x | x | x | x | Pöysä et al., 2023 |
| **Patajärvi** | 35 | *63° 15,187'* | *27° 10,348'* | 1985 | 2022 | 38 | 38 | 12 | x | x | x | x | Pöysä et al., 2023 |
| **Hämeenlahti** | 36 | *63° 9,524'* | *27° 22,278'* | 1985 | 2022 | 38 | 38 | 11 | x | x | x | x | Pöysä et al., 2023 |

**Table S3**. Species traits used in analyses.

| **Species** | **Body mass ^1)^** | **Age at 1^st^ reproduction ^2)^** | **Clucth size ^3)^** | **STI ^4)^** | **STR ^4)^** |
| --- | --- | --- | --- | --- | --- |
| *Podiceps cristatus* | 1059 | 2 | 3.5 | 10.1 | 24.7 |
| *Podiceps grisegena* | 954 | 2 | 3.5 | 2.3 | 8 |
| *Podiceps auritus* | 405 | 2 | 3.9 | 3.4 | 9.1 |
| *Cygnus cygnus* | 7965 | 4 | 5 | -1.5 | 12.5 |
| *Mareca penelope* | 581 | 1 | 7.7 | 16.5 | 23.2 |
| *Anas acuta* | 593 | 1 | 7.7 | 16.9 | 23.7 |
| *Anas platyrhynchos* | 1088 | 1 | 8.1 | 0 | 22.4 |
| *Anas crecca* | 277 | 1 | 8.1 | 12.7 | 25.2 |
| *Spatula querquedula* | 379 | 1 | 7.1 | 23.1 | 8.3 |
| *Spatula clypeata* | 527 | 1 | 8.9 | 14.9 | 23.3 |
| *Bucephala clangula* | 608 | 2 | 8.5 | -1.1 | 11.1 |
| *Mergus merganser* | 1196 | 2 | 9.4 | -0.4 | 7.9 |
| *Aythya fuligula* | 690 | 1 | 9.2 | 10.5 | 29.4 |
| *Aythya ferina* | 755 | 1 | 7.4 | 11.3 | 24.5 |
| *Fulica atra* | 662 | 1.5 | 6.3 | 5.9 | 16 |
|  |  |  |  |  |  |

Sources: 1) Piha et al. (2018); 2) Cramp and Simmons (1977, 1980); 3) Lehikoinen et al. (2011); 4) Gaget et al. (2020).

**Table S4**. General linear mixed-effects models for the overall importance of wintering conditions in driving population dynamics (growth rate) of different waterbird species. Population growth rate was the response variable and severity of the previous winter (WSI), Year and population density in year t-1 (Density) were used as explanatory variables. Lake ID was included as a random factor in all models.

| **Species** |  | **Predictor** | **Estimate** | **SE** | **t** | **p** |
| --- | --- | --- | --- | --- | --- | --- |
| *Podiceps cristatus* | | WSI | -0.001 | 0.001 | -1.419 | 0.157 |
|  |  | Year | -0.025 | 0.004 | -6.581 | 0.000 |
|  |  | Density | -0.780 | 0.068 | -11.499 | 0.000 |
|  |  |  |  |  |  |  |
| *Podiceps grisegena* | | WSI | 0.000 | 0.001 | -0.503 | 0.618 |
|  |  | Year | 0.009 | 0.005 | 1.717 | 0.093 |
|  |  | Density | -0.686 | 0.126 | -5.437 | 0.000 |
|  |  |  |  |  |  |  |
| *Podiceps auritus* | | WSI | -0.001 | 0.001 | -1.461 | 0.147 |
|  |  | Year | -0.005 | 0.004 | -1.328 | 0.187 |
|  |  | Density | -0.724 | 0.092 | -7.900 | 0.000 |
|  |  |  |  |  |  |  |
| *Cygnus cygnus* | | WSI | 0.000 | 0.000 | 0.302 | 0.763 |
|  |  | Year | 0.000 | 0.002 | 0.092 | 0.927 |
|  |  | Density | -0.69 | 0.090 | -7.646 | 0.000 |
|  |  |  |  |  |  |  |
| *Mareca penelope* | | WSI | 0.000 | 0.001 | 0.700 | 0.485 |
|  |  | Year | -0.013 | 0.003 | -4.226 | 0.000 |
|  |  | Density | -0.723 | 0.052 | -13.777 | 0.000 |
|  |  |  |  |  |  |  |
| *Anas acuta* | | WSI | 0.000 | 0.001 | 0.430 | 0.668 |
|  |  | Year | -0.024 | 0.006 | -3.745 | 0.000 |
|  |  | Density | -0.786 | 0.103 | -7.641 | 0.000 |
|  |  |  |  |  |  |  |
| *Anas platyrhynchos* | | WSI | -0.001 | 0.000 | -1.573 | 0.116 |
|  |  | Year | -0.001 | 0.002 | -0.591 | 0.555 |
|  |  | Density | -0.71 | 0.044 | -16.077 | 0.000 |
|  |  |  |  |  |  |  |
| *Anas crecca* | | WSI | 0.000 | 0.000 | -0.134 | 0.893 |
|  |  | Year | -0.007 | 0.002 | -2.750 | 0.006 |
|  |  | Density | -0.824 | 0.046 | -17.836 | 0.000 |
|  |  |  |  |  |  |  |
| *Spatula querquedula* | | WSI | -0.001 | 0.001 | -1.051 | 0.297 |
|  |  | Year | -0.028 | 0.007 | -3.853 | 0.000 |
|  |  | Density | -0.868 | 0.117 | -7.427 | 0.000 |
|  |  |  |  |  |  |  |
| *Spatula clypeata* | | WSI | 0.001 | 0.001 | 1.063 | 0.289 |
|  |  | Year | -0.006 | 0.003 | -1.700 | 0.091 |
|  |  | Density | -0.821 | 0.070 | -11.652 | 0.000 |
|  |  |  |  |  |  |  |
| *Bucephala clangula* | | WSI | 0.000 | 0.000 | -0.227 | 0.820 |
|  |  | Year | -0.005 | 0.002 | -2.537 | 0.011 |
|  |  | Density | -0.561 | 0.040 | -13.905 | 0.000 |
|  |  |  |  |  |  |  |
| *Aythya fuligula* | | WSI | 0.000 | 0.001 | 0.433 | 0.666 |
|  |  | Year | -0.035 | 0.008 | -4.290 | 0.000 |
|  |  | Density | -0.647 | 0.094 | -6.858 | 0.000 |
|  |  |  |  |  |  |  |
| *Aythya ferina* | | WSI | -0.001 | 0.001 | -0.867 | 0.390 |
|  |  | Year | -0.037 | 0.008 | -4.497 | 0.000 |
|  |  | Density | -0.558 | 0.125 | -4.457 | 0.000 |
|  |  |  |  |  |  |  |
| *Fulica atra* | | WSI | -0.002 | 0.001 | -2.833 | 0.005 |
|  |  | Year | -0.022 | 0.005 | -4.597 | 0.000 |
|  |  | Density | -0.358 | 0.066 | -5.455 | 0.000 |
|  |  |  |  |  |  |  |

**Table S5**. General linear mixed-effects models for comparing population growth rates between years after exceptionally cold winters and years after exceptionally mild winters. Winter type ‘mild’ was included in the intercept; ‘Estimate’ for variable ‘WSI’ is exceptionally cold winters compared with exceptionally mild winters.

| **Species** |  | **Predictor** | **Estimate** | **SE** | **t** | **p** |
| --- | --- | --- | --- | --- | --- | --- |
| *Podiceps grisegena* | | Intercept | 0.399 | 0.135 | 2.961 | 0.011 |
|  |  | WSI | -0.055 | 0.106 | -0.517 | 0.610 |
|  |  | Density | -0.434 | 0.111 | -3.894 | 0.001 |
|  |  |  |  |  |  |  |
| *Podiceps auritus* | | Intercept | 0.188 | 0.104 | 1.813 | 0.085 |
|  |  | WSI | -0.087 | 0.106 | -0.825 | 0.414 |
|  |  | Density | -0.302 | 0.087 | -3.48 | 0.001 |
|  |  |  |  |  |  |  |
| *Cygnus cygnus* | | Intercept | 0.422 | 0.075 | 5.651 | 0.000 |
|  |  | WSI | -0.001 | 0.046 | -0.032 | 0.974 |
|  |  | Density | -0.644 | 0.095 | -6.784 | 0.000 |
|  |  |  |  |  |  |  |
| *Mareca penelope* | | Intercept | 0.721 | 0.130 | 5.562 | 0.000 |
|  |  | WSI | 0.106 | 0.087 | 1.218 | 0.225 |
|  |  | Density | -0.725 | 0.074 | -9.751 | 0.000 |
|  |  |  |  |  |  |  |
| *Anas acuta* | | Intercept | 0.433 | 0.146 | 2.965 | 0.009 |
|  |  | WSI | -0.017 | 0.137 | -0.124 | 0.902 |
|  |  | Density | -0.366 | 0.095 | -3.860 | 0.000 |
|  |  |  |  |  |  |  |
| *Anas platyrhynchos* | | Intercept | 0.962 | 0.124 | 7.768 | 0.000 |
|  |  | WSI | -0.078 | 0.070 | -1.110 | 0.269 |
|  |  | Density | -0.713 | 0.067 | -10.693 | 0.000 |
|  |  |  |  |  |  |  |
| *Anas crecca* | | Intercept | 0.742 | 0.139 | 5.320 | 0.000 |
|  |  | WSI | 0.044 | 0.082 | 0.539 | 0.590 |
|  |  | Density | -0.504 | 0.072 | -7.031 | 0.000 |
|  |  |  |  |  |  |  |
| *Spatula querquedula* | | Intercept | 0.572 | 0.142 | 4.022 | 0.001 |
|  |  | WSI | -0.209 | 0.121 | -1.728 | 0.090 |
|  |  | Density | -0.673 | 0.102 | -6.607 | 0.000 |
|  |  |  |  |  |  |  |
| *Spatula clypeata* | | Intercept | 0.509 | 0.148 | 3.442 | 0.002 |
|  |  | WSI | 0.067 | 0.109 | 0.619 | 0.538 |
|  |  | Density | -0.506 | 0.094 | -5.351 | 0.000 |
|  |  |  |  |  |  |  |
| *Bucephala clangula* | | Intercept | 0.515 | 0.086 | 5.979 | 0.000 |
|  |  | WSI | 0.121 | 0.063 | 1.916 | 0.057 |
|  |  | Density | -0.357 | 0.050 | -7.093 | 0.000 |
|  |  |  |  |  |  |  |
| *Mergus merganser* | | Intercept | 0.528 | 0.106 | 4.983 | 0.000 |
|  |  | WSI | -0.09 | 0.127 | -0.708 | 0.484 |
|  |  | Density | -0.956 | 0.122 | -7.826 | 0.000 |
|  |  |  |  |  |  |  |
| *Aythya fuligula* | | Intercept | 0.476 | 0.099 | 4.798 | 0.000 |
|  |  | WSI | -0.061 | 0.122 | -0.500 | 0.619 |
|  |  | Density | -0.405 | 0.075 | -5.416 | 0.000 |
|  |  |  |  |  |  |  |
| *Aythya ferina* | | Intercept | 0.424 | 0.152 | 2.788 | 0.010 |
|  |  | WSI | -0.088 | 0.130 | -0.677 | 0.502 |
|  |  | Density | -0.445 | 0.099 | -4.492 | 0.000 |
|  |  |  |  |  |  |  |
| *Fulica atra* | | Intercept | 0.534 | 0.149 | 3.586 | 0.002 |
|  |  | WSI | -0.593 | 0.132 | -4.506 | 0.000 |
|  |  | Density | -0.207 | 0.066 | -3.127 | 0.003 |
|  |  |  |  |  |  |  |


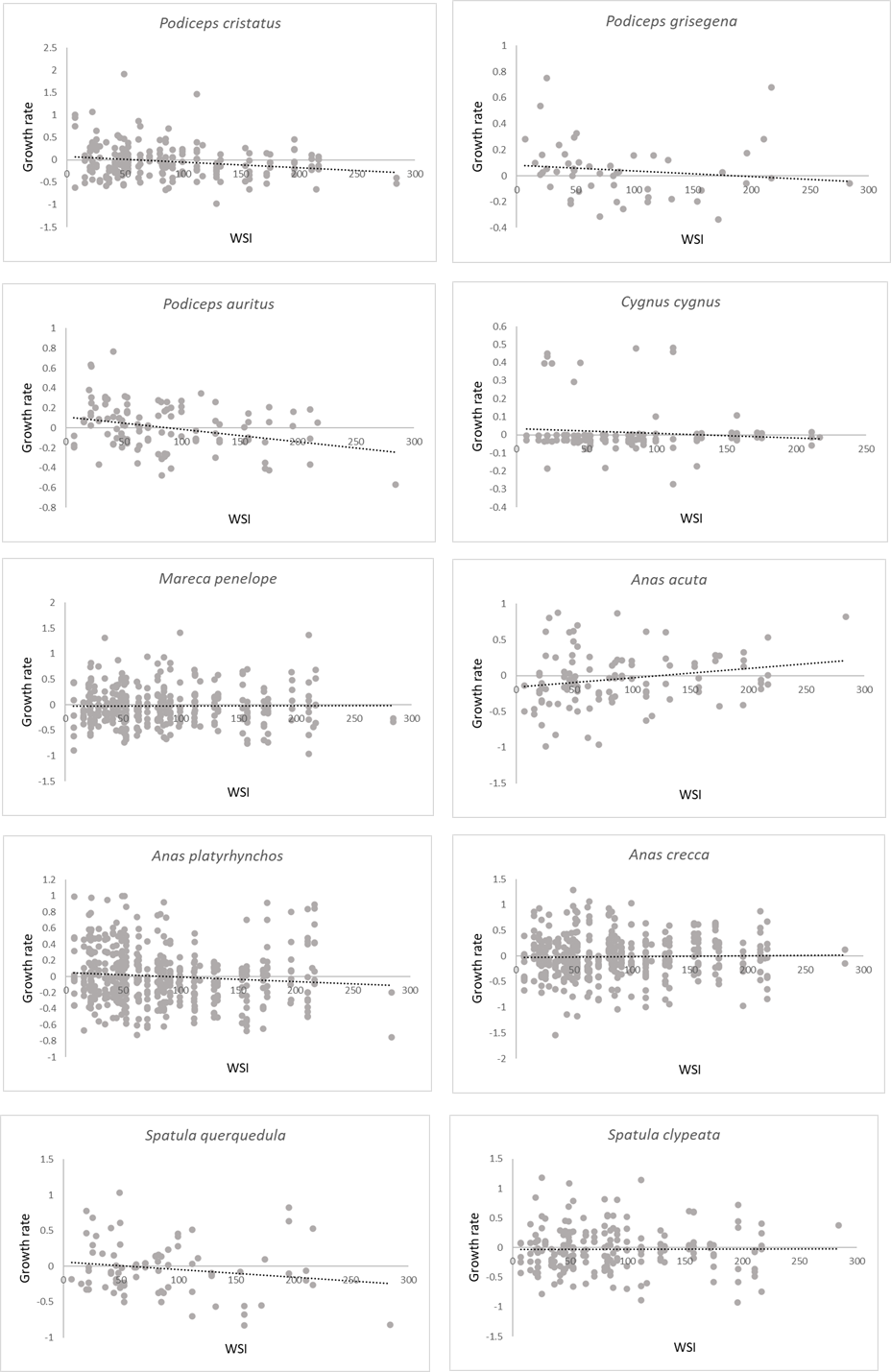


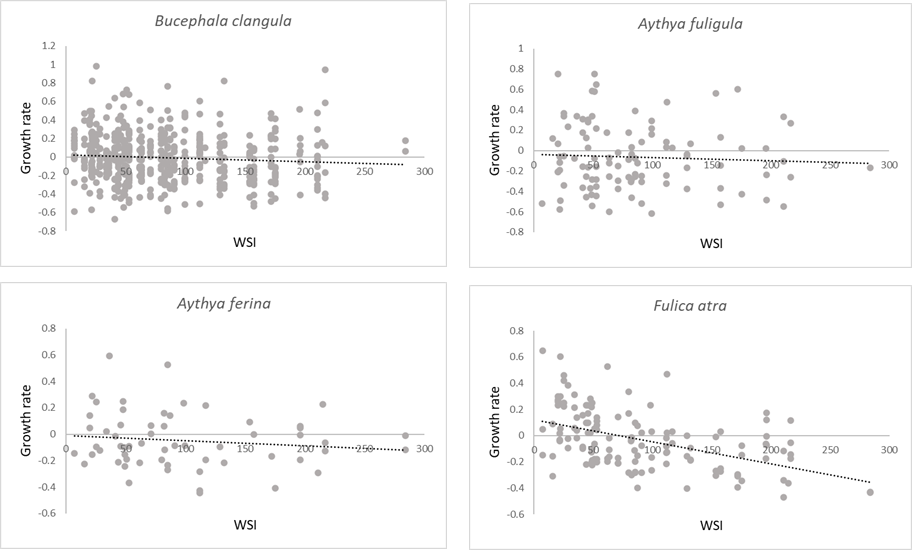


**Fig. S3**. Effect of winter severity (WSI) on population growth rate for different waterbird species. Figures are drawn based on predicted values from species-specific models presented in Table S4. Trend lines (dashed lines) are drawn for illustrative purposes (see Table S4 for statistical evidence of the dependence of growth rate of WSI and the main text for further details).


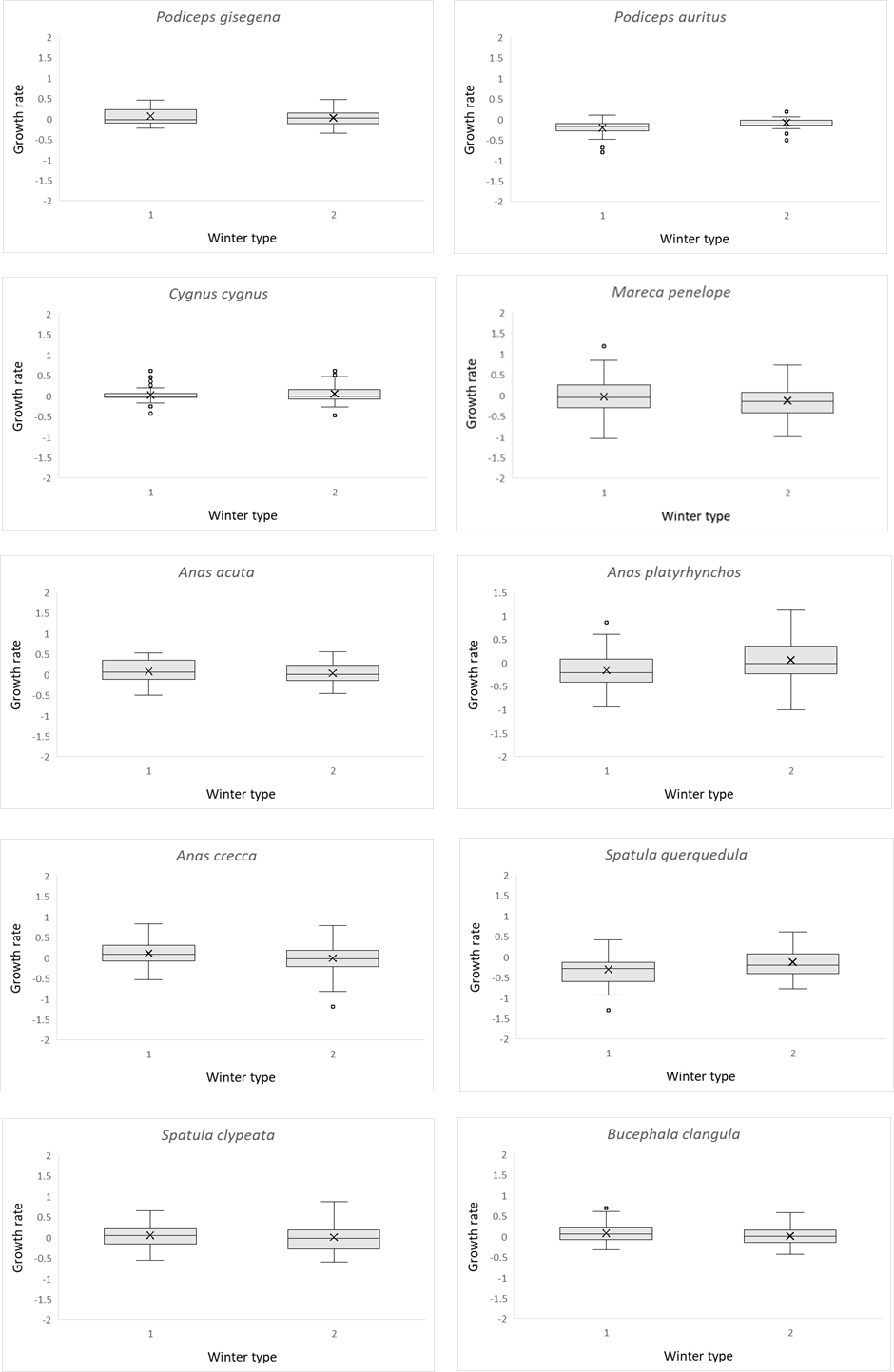


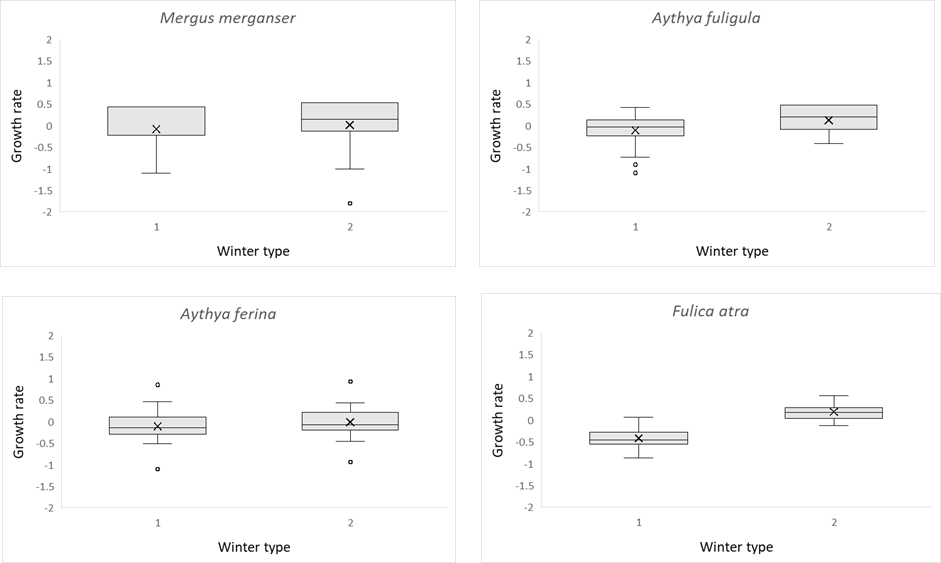


**Fig. S4**. Population growth rates in years after exceptionally cold winters (winter type 1) and in years after exceptionally mild winters (winter type 2). Figures are drawn based on predicted values from species-specific models presented in Table S5.

**Table S6**. Linear regressions between standardized annual pair number and year (time) in 1987–1991 for different waterbird species to examine population recovery after three consecutive exceptionally cold winters.

| **Species** |  | **Predictor** | **Estimate** | **SE** | **t** | **p** | **n** |
| --- | --- | --- | --- | --- | --- | --- | --- |
| *Podiceps cristatus* | | Intercept | 0.418 | 0.281 | 1.488 | 0.143 |  |
|  |  | Time | -0.139 | 0.085 | -1.645 | 0.106 | 55 |
|  |  |  |  |  |  |  |  |
| *Podiceps grisegena* | | Intercept | -1.314 | 0.535 | -2.457 | 0.040 |  |
|  |  | Time | 0.438 | 0.161 | 2.716 | 0.026 | 10 |
|  |  |  |  |  |  |  |  |
| *Podiceps auritus* | | Intercept | -0.631 | 0.466 | -1.353 | 0.193 |  |
|  |  | Time | 0.210 | 0.141 | 1.496 | 0.152 | 20 |
|  |  |  |  |  |  |  |  |
| *Mareca penelope* | | Intercept | -0.210 | 0.253 | -0.83 | 0.409 |  |
|  |  | Time | 0.070 | 0.076 | 0.918 | 0.362 | 70 |
|  |  |  |  |  |  |  |  |
| *Anas acuta* | | Intercept | -0.473 | 0.310 | -1.526 | 0.134 |  |
|  |  | Time | 0.158 | 0.093 | 1.687 | 0.099 | 45 |
|  |  |  |  |  |  |  |  |
| *Anas platyrhynchos* | | Intercept | -0.518 | 0.221 | -2.340 | 0.022 |  |
|  |  | Time | 0.173 | 0.067 | 2.587 | 0.011 | 85 |
|  |  |  |  |  |  |  |  |
| *Anas crecca* | | Intercept | -0.604 | 0.218 | -2.769 | 0.007 |  |
|  |  | Time | 0.201 | 0.066 | 3.063 | 0.003 | 85 |
|  |  |  |  |  |  |  |  |
| *Spatula querquedula* | | Intercept | -0.486 | 0.279 | -1.746 | 0.087 |  |
|  |  | Time | 0.162 | 0.084 | 1.930 | 0.059 | 55 |
|  |  |  |  |  |  |  |  |
| *Spatula clypeata* | | Intercept | -0.464 | 0.255 | -1.820 | 0.074 |  |
|  |  | Time | 0.155 | 0.077 | 2.012 | 0.049 | 60 |
|  |  |  |  |  |  |  |  |
| *Bucephala clangula* | | Intercept | -0.835 | 0.247 | -3.375 | 0.001 |  |
|  |  | Time | 0.278 | 0.075 | 3.731 | 0.000 | 60 |
|  |  |  |  |  |  |  |  |
| *Aythya fuligula* | | Intercept | -0.078 | 0.254 | -0.306 | 0.761 |  |
|  |  | Time | 0.026 | 0.077 | 0.338 | 0.736 | 70 |
|  |  |  |  |  |  |  |  |
| *Aythya ferina* | | Intercept | 0.227 | 0.273 | 0.831 | 0.409 |  |
|  |  | Time | -0.076 | 0.082 | -0.919 | 0.362 | 60 |
|  |  |  |  |  |  |  |  |
| *Fulica atra* | | Intercept | -1.269 | 0.271 | -4.676 | 0.000 |  |
|  |  | Time | 0.423 | 0.082 | 5.169 | 0.000 | 35 |
|  |  |  |  |  |  |  |  |

**Table S7**. Long-term population trends (Slope) for different waterbird species as indicated by slopes of linear regressions between standardized annual pair number and year in 1987–2022 (n = 36 in all cases). Species’ long-term population trends (percentage change of breeding numbers during 1986–2025, last column) based on the Finnish national monitoring data are also given (from Piha et al., 2025). Species’ population trend indices are strongly correlated between the two data sets (Slope vs. Percentage population change, Kendall rank correlation, τ = 0.692, p = 0.0007, n = 14).

.

| **Species** |  | **Slope** | **SE** | **t** | **p** | **Percentage population change based on Finnish monitoring data** |
| --- | --- | --- | --- | --- | --- | --- |
| *Podiceps cristatus* | | -0.079 | 0.009 | -8.789 | 0.000 | -54 |
|  |  |  |  |  |  |  |
| *Podiceps grisegena* | | 0.043 | 0.015 | 2.961 | 0.006 | -38 |
|  |  |  |  |  |  |  |
| *Podiceps auritus* | | -0.076 | 0.010 | -7.701 | 0.000 | -80 |
|  |  |  |  |  |  |  |
| *Cygnus cygnus* | | 0.086 | 0.007 | 12.255 | 0.000 | +294 |
|  |  |  |  |  |  |  |
| *Mareca penelope* | | -0.071 | 0.011 | -6.493 | 0.000 | -61 |
|  |  |  |  |  |  |  |
| *Anas acuta* | | -0.073 | 0.010 | -7.030 | 0.000 | -75 |
|  |  |  |  |  |  |  |
| *Anas platyrhynchos* | | 0.025 | 0.016 | 1.569 | 0.126 | +15 |
|  |  |  |  |  |  |  |
| *Anas crecca* | | -0.019 | 0.016 | -1.208 | 0.235 | -26 |
|  |  |  |  |  |  |  |
| *Spatula querquedula* | | -0.069 | 0.011 | -6.216 | 0.000 | NA |
|  |  |  |  |  |  |  |
| *Spatula clypeata* | | -0.022 | 0.016 | -1.398 | 0.171 | -69 |
|  |  |  |  |  |  |  |
| *Bucephala clangula* | | -0.027 | 0.016 | -1.757 | 0.088 | -13 |
|  |  |  |  |  |  |  |
| *Mergus merganser* | | -0.021 | 0.016 | -1.323 | 0.195 | -45 |
|  |  |  |  |  |  |  |
| *Aythya fuligula* | | -0.084 | 0.008 | -11.004 | 0.000 | -78 |
|  |  |  |  |  |  |  |
| *Aythya ferina* | | -0.088 | 0.006 | -15.035 | 0.000 | -97 |
|  |  |  |  |  |  |  |
| *Fulica atra* | | -0.080 | 0.009 | -9.196 | 0.000 | -74 |
|  |  |  |  |  |  |  |

**References**

Cramp, S., Simmons, K.E.L. (Eds.), 1977. Birds of the Western Palearctic,Vol. 1. Oxford University Press, Oxford.

Cramp, S., Simmons, K.E.L. (Eds.), 1980. Birds of the Western Palearctic, Vol. 2. Oxford University Press, Oxford.

Gaget, E., Galewski, T., Jiguet, F., Guelmami, A., Perennou, C., Beltrame, C., Le Viol, I., 2020. Antagonistic effect of natural habitat conversion on community adjustment to climate warming in nonbreeding waterbirds. Conserv. Biol. 34, 966–976.

Kauppinen, J., Väänänen, V.-M., 1999. Factors affecting changes in waterfowl populations in eutrophic wetlands in the Finnish lake district. Wildl. Biol. 5, 73–81.

Klein Tank, A.M.G., Wijngaard, J., Können, G.P., Böhm, R., Demarée, G., Gocheva, A., Mileta, M., Pashiardis, S., Hejkrlik, L., Kern-Hansen, C., Heino, R., Bessemoulin, P., Müller-Westermeier, G., Tzanakou, M., Szalai, S., Pálsdóttir, T., Fitzgerald, D., Rubin, S., Capaldo, M., Maugeri, M., Leitass, A,., Bukantis, A., Aberfeld, R., van Engelen, A.F.V., Forland, E., Mietus, M., Coelho, F., Mares, C., Razuvaev, V., Nieplova, E., Cegnar, T., Antonio, López, J., Dahlström, B., Moberg, A., Kirchhofer, W., Ceylan, A., Pachaliuk, O., Alexander, L.V., Petrovic, P., 2002. Daily dataset of 20th-century surface air temperature and precipitation series for the European Climate Assessment. Int. J. Climatol 22, 1441–1453.

Lehikoinen, A., Honkala, J., Piirainen, E., 2011. Kuinka monta munaa on linnun pesässä? Suomen pesivien lintujen munaluku seuranta-aineistojen perusteella. Linnut-vuosikirja 2011, 144–150.

Piha, M., Valkama, J., Lehikoinen, E., 2018. Suomen lintujen painot ja siipien pituudet – osa 2: ei-varpuslinnut (Summary: Body mass and wing length of birds based on the Finnish ringing data base – Part 2: non-passerines and corvids). Linnut-vuosikirja 2018, 166–175.

Piha, M., Ikonen, K., Lindén, A., Lehikoinen, A., Rajala, T., Seimola, T. 2025. Finnish waterfowl monitoring results 1986–2025. Luonnonvara- ja biotalouden tutkimus 89/2025. Luonnonvarakeskus. Helsinki. 21 p.

Pöysä, H., Linkola, P., 2021. Extending temporal baseline increases understanding of biodiversity change in European boreal waterbird communities. Biol. Conserv. 257, 109139.

Pöysä, H., Lammi, E., Pöysä, S., Väänänen, V.-M., 2023. When good turns to bad and alien predators appear: the dynamics of biodiversity change in boreal waterbird communities. Global Ecol. Conserv. 48, e02727.

Tossavainen, M., Tossavainen, T., 1984. Vetelin Räyringinjärven vesi- ja rantalinnustosta. Ornis Botnica 6, 2‒13.

Virta, H., 1985. Tuntematon lintuharrastajan aarre ‒ Nurmesjärvi. Aureola 10, 83‒87.
